# A multi-locus approach to barcoding in the *Anopheles strodei* subgroup (Diptera: Culicidae)

**DOI:** 10.1186/1756-3305-6-111

**Published:** 2013-04-19

**Authors:** Brian Patrick Bourke, Tatiane Porangaba Oliveira, Lincoln Suesdek, Eduardo Sterlino Bergo, Maria Anice Mureb Sallum

**Affiliations:** 1 Departamento de Epidemiologia, Faculdade de Saúde Pública, Universidade de São Paulo, São Paulo-SP, Brazil; 2 Laboratório de Parasitologia, Instituto Butantan, Avenida Vital Brasil, São Paulo-SP, Brazil; 3 Superintendência de Controle de Endemias, Secretaria de Estado da Saúde de São Paulo, Araraquara-SP, Brazil

**Keywords:** Culicidae, *Anopheles*, Strodei Subgroup, Identification, Barcode, *COI*, ITS2, *White*, Atlantic Forest, New species

## Abstract

**Background:**

The ability to successfully identify and incriminate pathogen vectors is fundamental to effective pathogen control and management. This task is confounded by the existence of cryptic species complexes. Molecular markers can offer a highly effective means of species identification in such complexes and are routinely employed in the study of medical entomology. Here we evaluate a multi-locus system for the identification of potential malaria vectors in the *Anopheles strodei* subgroup.

**Methods:**

Larvae, pupae and adult mosquitoes (n = 61) from the *An. strodei* subgroup were collected from 21 localities in nine Brazilian states and sequenced for the *COI*, ITS2 and *white* gene. A Bayesian phylogenetic approach was used to describe the relationships in the Strodei Subgroup and the utility of *COI* and ITS2 barcodes was assessed using the neighbor joining tree and “best close match” approaches.

**Results:**

Bayesian phylogenetic analysis of the *COI*, ITS2 and *white* gene found support for seven clades in the *An*. *strodei* subgroup. The *COI* and ITS2 barcodes were individually unsuccessful at resolving and identifying some species in the Subgroup. The *COI* barcode failed to resolve *An*. *albertoi* and *An*. *strodei* but successfully identified approximately 92% of all species queries, while the ITS2 barcode failed to resolve *An*. *arthuri* and successfully identified approximately 60% of all species queries. A multi-locus *COI*-ITS2 barcode, however, resolved all species in a neighbor joining tree and successfully identified all species queries using the “best close match” approach.

**Conclusions:**

Our study corroborates the existence of *An*. *albertoi*, *An*. CP Form and *An*. *strodei* in the *An. strodei* subgroup and identifies four species under *An*. *arthuri* informally named A-D herein. The use of a multi-locus barcode is proposed for species identification, which has potentially important utility for vector incrimination. Individuals previously found naturally infected with *Plasmodium vivax* in the southern Amazon basin and reported as *An*. *strodei* are likely to have been from *An*. *arthuri* C identified in this study.

## Background

One of the most important goals of medical entomology is to develop approaches that effectively identify the roles of insect species in transmitting infectious pathogens. The incrimination of a pathogen vector requires demonstrating that the species feeds on humans, an association in time and space between the species and the occurrence of human infections, repeated isolation of the pathogen from the species, and the transmission of the pathogen by the species under controlled experimental conditions [1]. Fundamental to the process of incrimination is an ability to resolve and identify species effectively. However, many vector species are morphologically indistinguishable from close relatives yet they can exhibit a range of genetic, biological and morphological variation [2]. Such species form cryptic species complexes and their existence makes the task of vector incrimination more difficult. Molecular approaches are now routinely used to help resolve such complexes and have become essential tools in the study of medical entomology and infectious disease transmission.

The phylogenetic analysis of species complexes employs markers with relatively high rates of substitution that are likely to track recently diverged species. A multi-locus approach can reconstruct more robust evolutionary relationships, discover previously unknown lineages in species and inform the search for latent morphological differences. Recently, DNA barcoding initiatives have proposed approaches that employ “sequence diversity in short, standardized gene regions to aid species identification and discovery in large assemblages of life” [3]. Various molecular markers [4-6] have been employed but it is cytochrome c oxidase I (*COI*) that has gained acceptance as the “gold standard” barcode for animals. The internal transcribed spacer region 2 (ITS2) has also been employed as a barcode region, primarily for plants but increasingly for animals [5]. The success of the barcoding approach is related to inter-specific variation exceeding intra-specific variation (the existence of the “barcoding gap”), and the analysis to date has generally been performed using clustering (neighbor joining tree monophyly) or pairwise genetic distances [7]. Recently diverged or incipient species, however, may be frequently misidentified due to incomplete lineage sorting of ancestral polymorphisms [8-10]. While barcoding is therefore a useful approach to determine minimum estimates of species numbers in cryptic species complexes although see [11], multi-locus and multi-data (genetic/morphological/ecological) approaches are likely to be more effective at elucidating the full extent of species diversity within these systems.

The current study focuses on species diversity within the Neotropical Strodei Subgroup of *Anopheles* (*Nyssorhynchus*) mosquitoes. This Subgroup is currently comprised of five species (*Anopheles albertoi* Unti*, Anopheles arthuri* Unti*, Anopheles* CP Form [12]*, Anopheles rondoni* (Neiva and Pinto) and *Anopheles strodei* Root), which are distributed through much of Central and South America, from Panama to Argentina [13,14], although several additional taxa have been described and synonymized historically. *Anopheles strodei* was first described using morphological characters of the adult male, fourth-instar larvae and pupae from specimens from Juiz de Fora, Minas Gerais State, Brazil [15]. Later, *An. albertoi*, *An. arthuri*, *An. artigasi* Unti, and *An. lloydi* Unti were described based on egg characteristics and *Anopheles ramosi* Unti by the fourth-instar larvae [16,17]. The type localities of *An. albertoi*, *An. arthuri*, *An. artigasi*, *An. ramosi* are all from Vale do Paraíba, São Paulo state, Brazil, whereas that of *An. lloydi* is an unspecified location in Panama. Further examination of *An. strodei* based on adult female, larvae [13] and egg [18] morphology and patterns of the salivary polytene chromosome [19] showed high levels of polymorphism throughout its range and led Faran [13] to synonomize *An. strodei*, *An. albertoi*, *An. arthuri*, *An. artigasi*, *An. lloydi*, *An. ramosi* and *An. strodei* into a single species. A recent study of *COI* gene and *white* gene [12] sequences allowed the resurrection of *An. albertoi* and *An. arthuri* from synonomy with *An. strodei*, and revealed an undescribed taxon, preliminarily named *An.* CP Form.

Although Neotropical *Anopheles* species are known vectors of filariasis (*Wuchereria bancrofti* Cobbold [20]), arboviruses (Anopheles A Virus [21]) and malaria [22], the importance of the Strodei Subgroup in vectoring parasites is largely unknown. *Anopheles strodei*, however, has previously been found naturally infected with *Plasmodium vivax* Grassi & Feletti in Ariquemes, Rondônia, in the Amazon region, [23] although it remains unknown whether this record refers to *An*. *strodei* s.s. or another member of the Strodei Subgroup. The continental distribution of this complex confounds efforts to comprehensively describe species diversity and, ultimately, vectorial capacity. Our study seeks to provide a more complete understanding of species diversity and distribution in the Strodei Subgroup by performing a multi-locus DNA analysis of specimens collected from across Brazil. We will first resolve species relationships with a Bayesian approach using the *COI*, ITS2 and *white* gene. We will then test the utility of the *COI* barcode and the less frequently employed ITS2 barcode for species identification in the *An*. *strodei* subgroup.

## Methods

### Mosquito collection

Collection localities and identity of the specimens included in this study can be found in Table 1. These specimens were either offspring of females caught in the field using a Shannon trap or larvae and pupae collected from immature habitats, which were then raised to adulthood. Species identification of all but two specimens was based on adult male genitalia, fourth-instar larval characteristics or scanning electron micrographs of the egg. Individuals from *An*. *arthuri* displayed substantial variation in male genitalia and so were identified as *An*. *arthuri* sensu lato.

**Table 1:** Sample information, including specimen numbers, species, localities, geographical coordinates, and Genbank accession numbers

**Specimen**	**Species**	**Locality (state)**	**Latitude**	**Longitude**	**COI GenBank accession no.**	**ITS2 GenBank accession no.**	**White GenBank accession no.**
MG07 12 4	*Anopheles albertoi*	Frutal (Minas Gerais)	-20.025278	-49.076500	GU226678	FJ178885	GU226747
MG07 3 4	*Anopheles albertoi*	Frutal (Minas Gerais)	-20.025278	-49.076500	GU226676	FJ178889	GU226742
MG07 7 10	*Anopheles albertoi*	Frutal (Minas Gerais)	-20.025278	-49.076500	GU226677	FJ178886-FJ178888	GU226743-GU226746
CE12 1 1	*Anopheles arthuri* B	Ubajara (Ceará)	-3.8867500	-41.001250	KC330250	KC330268	KC330325
CE12 4 6	*Anopheles arthuri* B	Ubajara (Ceará)	-3.8867500	-41.001250	KC330251	KC330269	KC330326
CE17 15 2	*Anopheles arthuri* B	Ubajara (Ceará)	-3.8442220	-40.897778	KC330253	KC330270	KC330328
CE17 5 1	*Anopheles arthuri* B	Ubajara (Ceará)	-3.8442220	-40.897778	KC330252	KC330271	KC330327
CE20 10 4	*Anopheles arthuri* B	São Benedito (Ceará)	-4.0964170	-40.896361	KC330254	KC330272	KC330329
CE20 24-3	*Anopheles arthuri* B	São Benedito (Ceará)	-4.0964170	-40.896361	KC330255	KC330273	KC330330
GO7 1 3	*Anopheles arthuri* A	Itarumã (Goiás)	-18.906128	-51.024917	KC330244	KC330274	KC330319
GO7 2 102	*Anopheles arthuri* A	Itarumã (Goiás)	-18.906128	-51.024917	KC330245	KC330275-KC330277	KC330320
GO7 3 105	*Anopheles arthuri* A	Itarumã (Goiás)	-18.906128	-51.024917	KC330246	KC330278	KC330321
GO7 6 101	*Anopheles arthuri* A	Itarumã (Goiás)	-18.906128	-51.024917	KC330247	KC330279-KC330282	KC330322
MG03 102	*Anopheles arthuri* A	Frutal (Minas Gerais)	-19.981278	-49.096028	GU226679	FJ178881-FJ178884	GU226748
MG04 102	*Anopheles arthuri* A	Frutal (Minas Gerais)	-19.988472	-49.093361	GU226680	FJ178880	GU226751
MG07 1 100	*Anopheles arthuri* A	Frutal (Minas Gerais)	-20.025278	-49.076500	GU226683	FJ178879	GU226752
MG07 10 106	*Anopheles arthuri* A	Frutal (Minas Gerais)	-20.025278	-49.076500	GU226684	GU226712-GU226717	GU226756
MG07 18 100	*Anopheles arthuri* A	Frutal (Minas Gerais)	-20.025278	-49.076500	GU226685	GU226706	GU226753-GU226755
MG07 20 2	*Anopheles arthuri* A	Frutal (Minas Gerais)	-20.025278	-49.076500	GU226686	GU226707-GU226711	GU226750
MG07 6 3	*Anopheles arthuri* A	Frutal (Minas Gerais)	-20.025278	-49.076500	GU226681	GU226700-GU226705	GU226749
MG24 1	*Anopheles arthuri* A	Goianá (Minas Gerais)	-21.538836	-43.200856	GU226682	GU226723-GU226725	GU226757
MG32 4	*Anopheles arthuri* A	Oliveira (Minas Gerais)	-20.746389	-44.915278	KC330257	KC330283	KC330332
MG33 11 2	*Anopheles arthuri* A	Oliveira (Minas Gerais)	-20.745598	-44.915613	KC330258	KC330284	KC330340
MG33 12 6	*Anopheles arthuri* A	Oliveira (Minas Gerais)	-20.745598	-44.915613	KC330259	KC330285	KC330334
MG33 13 7	*Anopheles arthuri* D	Oliveira (Minas Gerais)	-20.508785	-44.770600	KC330256	KC330286-KC330288	KC330331
MG34 2	*Anopheles arthuri* A	Oliveira (Minas Gerais)	-20.712500	-44.974444	KC330260	KC330289	KC330335
MG34 9	*Anopheles arthuri* A	Oliveira (Minas Gerais)	-20.712500	-44.974444	KC330261	KC330290	KC330336
MG35 11	*Anopheles arthuri* D	São Franscisco de Paula (Minas Gerais)	-20.754444	-44.917222	KC330262	KC330291	KC330338
MG44 14 2	*Anopheles arthuri* A	Oliveira (Minas Gerais)	-20.768428	-44.878209	KC330263	KC330292	KC330339
RO29 18	*Anopheles arthuri* C	Campo Novo de Rondônia (Rondônia)	-10.637639	-65.499833	KC330248	KC330293	KC330323
RO31 103	*Anopheles arthuri* C	Campo Novo de Rondônia (Rondônia)	-10.637639	-65.499833	KC330249	KC330294	KC330324
RO8 1	*Anopheles arthuri* C	Monte Negro (Rondônia)	-10.268639	-63.555389	GU226681	GU226727	GU226759
RO8 104	*Anopheles arthuri* C	Monte Negro (Rondônia)	-10.268639	-63.555389	GU226690	GU226728	GU226760
RO8 109	*Anopheles arthuri* C	Monte Negro (Rondônia)	-10.268639	-63.555389	GU226689	GU226729	GU226761
SP31 120	*Anopheles arthuri* A	Inubia Paulista (São Paulo)	-21.681417	-50.919889	GU226687	GU226699, GU226718-GU226722	GU226758
MG15 1 1	*Anopheles* CP Form	Coronel Pacheco (Minas Gerais)	-21.635819	-43.319267	JN413711	KC330265	KC330316
MG15 6 12	*Anopheles* CP Form	Coronel Pacheco (Minas Gerais)	-21.635819	-43.319267	JN413712	KC330266	KC330317
MG15 9 6	*Anopheles* CP Form	Coronel Pacheco (Minas Gerais)	-21.635819	-43.319267	KC330243	KC330267	KC330318
PR21 110	*Anopheles* CP Form	Foz do Iguaçu (Paraná)	-54.546528	-25.454583	GU226691	FJ178890	GU226762
BA23 3	*Anopheles strodei*	São José da Vitória (Bahia)	-15.087060	-39.341560	KC330234	KC330296	KC330308
BA25 4	*Anopheles strodei*	São José da Vitória (Bahia)	-15.090910	-39.343700	KC330235	KC330297	KC330309
ES09 1	*Anopheles strodei*	Santa Teresa (Espírito Santo)	-19.916667	-40.600000	GU226664	FJ178875	GU226730
ES09 3	*Anopheles strodei*	Santa Teresa (Espírito Santo)	-19.916667	-40.600000	GU226665	FJ178874	GU226731
MG27 108	*Anopheles strodei*	Coronel Pacheco (Minas Gerais)	-21.587778	-43.265833	GU226669	GU226693	GU226735
MG30 102	*Anopheles strodei*	Coronel Pacheco (Minas Gerais)	-21.587778	-43.265834	GU226670	GU226694	GU226736
MG33 9 1	*Anopheles strodei*	Oliveira (Minas Gerais)	-20.745598	-44.915613	KC330242	KC330298	KC330333
PR20 4 3	*Anopheles strodei*	São Miguel do Iguaçu (Paraná)	-25.265361	-54.309583	KC330233	KC330299	KC330307
PR29 23 3	*Anopheles strodei*	Foz do Iguaçu (Paraná)	-25.480556	-54.586667	GU226671	GU226695	GU226737
RS37 9 8	*Anopheles strodei*	Maquiné (Rio Grande do Sul)	-29.589556	-50.262639	KC330236	KC330300	KC330310
SP07 6	*Anopheles strodei*	Buri (São Paulo)	-23.800000	-48.566670	GU226674	FJ178878	GU226740
SP104 18 1	*Anopheles strodei*	Pindamonhangaba (São Paulo)	-22.960472	-45.452083	KC330240	KC330301	KC330314
SP105 10 12	*Anopheles strodei*	Pindamonhangaba (São Paulo)	-22.999333	-45.495361	KC330241	KC330302	KC330315
SP27 1	*Anopheles strodei*	Lucélia (São Paulo)	-21.618861	-50.940000	GU226672	GU226696	GU226738
SP29 121	*Anopheles strodei*	Lucélia (São Paulo)	-21.618861	-50.940000	GU226675	GU226698	GU226741
SP31 101	*Anopheles strodei*	Inubia Paulista (São Paulo)	-21.681417	-50.919889	KC330232	GU226697	KC330306
SP56 33	*Anopheles strodei*	Mairiporã (São Paulo)	-23.318889	-46.586944	KC330238	KC330303	KC330312
SP56 8	*Anopheles strodei*	Mairiporã (São Paulo)	-23.318889	-46.586944	KC330237	KC330304	KC330311
SP66 15 1	*Anopheles strodei*	Dourado (São Paulo)	-22.134694	-48.391722	KC330239	KC330305	KC330313
VP05 11A	*Anopheles strodei*	Pindamonhangaba (São Paulo)	-22.959750	-45.452389	GU226668	FJ178877	GU226734
VP06 5 2	*Anopheles strodei*	Pindamonhangaba (São Paulo)	-22.959750	-45.452389	GU226667	FJ178876	GU226733
VP06 6 4	*Anopheles strodei*	Pindamonhangaba (São Paulo)	-22.959750	-45.452389	GU226666	GU226692	GU226732

### DNA Extraction

DNA was extracted from each specimen according to the animal tissue DNA extraction protocol provided by the QIAgen DNeasy^®^ Blood and Tissue Kit (QIAgen Ltd, Crawley, UK). All extractions were diluted to 200 μL with the buffer provided and extraction solutions were retained for storage at −80°C in the entomological frozen collection of the Faculdade de Saúde Pública, Universidade de São Paulo, Brazil.

### *COI* gene

The gene was amplified using LCO- 1490 (5′-GGT CAA CAA ATC ATA AAG ATA TTG G-3′) and HCO-2198 (5′-TAA ACT TCA GGG TGA CCA AAA ATC A-3′) primers [24]. The Polymerase Chain Reaction (PCR) was carried out in a 25-μL aqueous reaction mixture containing 1 μL of DNA extraction solution, 1X PCR buffer (Invitrogen, Carlsbad, CA, USA), 1.5 mM MgCl_2_ (Invitrogen), 1.25 μL dimethly sulfoxide (Sigma, St. Louis, MO, USA), 0.1 μM of each primer, 0.2 mM each dNTP (Amresco, Solon, OH, USA) and 1.25 U Taq Platinum polymerase (Invitrogen). The reaction proceeded under the following temperature profile: 95°C for 2 min, 35 cycles of 94°C for 1 min, 57°C for 1 min and 72°C for 1 min and a final extension at 72°C for 7 min.

### ITS2 region

This region was amplified using 5.8SF (5′-ATC ACT CGG CTC GTG GAT CG-3′) and 28SR (5′-ATG CTT AAA TTT AGG GGG TAG TC-3′) primers [25]. The PCR was carried out in a 25-μL aqueous reaction mixture containing 1 μL of DNA extraction solution, 1X PCR buffer (Invitrogen), 1.5 mM MgCl_2_ (Invitrogen), 1.25 μL dimethyl sulfoxide (Sigma), 0.1 μM of each primer, 0.2 mM each dNTP (Amresco) and 1.25 U Taq Platinum polymerase (Invitrogen). The reaction proceeded under the following temperature profile: 94°C for 2 min, 34 cycles of 94°C for 30 s, 57°C for 30 s and 72°C for 30 s and a final extension at 72°C for 10 min. ITS2 amplicons that yielded ambiguous sequence chromatograms, which is suggestive of intragenomic variation, were purified using PEG precipitation (20% polyethylene glycol 8,000/2.5 M NaCl) and then cloned into pGem-T Easy Vector (Promega, Madison, WI).

### *White* gene

This gene was amplified using WZ2E and WZ11 primers [26]. This amplification product then served as a template in a sequencing reaction using internal primers W1F (5′-GAT CAA RAA GAT CTG YGA CTC GTT-3′) and W2R (5′GCC ATC GAG ATG GAG GAG CTG-3′). Both PCRs were carried out in a 25-μL aqueous reaction mixture containing 1 μl DNA extraction solution, 1X PCR buffer (Invitrogen), 1.5 mM MgCl_2_ (Invitrogen), 2.5 μL of dimethyl sulfoxide (Sigma), 2.0 μM of each primer, 0.2 mM each dNTP (Amresco) and 2.5 U Taq Platinum polymerase (Invitrogen). Both PCRs proceeded under the following temperature profile: 94°C for 5 min, 35 cycles at 94°C for 30 s, an annealing temperature of 50°C for 1 min and then 72°C for 2 min followed by a final extension at 72°C for 10 min. Any *white* amplicons that yielded ambiguous sequence chromatograms were purified using PEG precipitation (20% polyethylene glycol 8,000/2.5 M NaCl) and then cloned into pGem-T Easy Vector (Promega).

### Sequencing and sequence alignment

Sequencing reactions were carried out in both directions using a Big Dye Terminator cycle sequencing kit v3.1 (Applied Biosystems, Foster City, CA, USA) and Applied Biosystems 3130 DNA Analyzer (Applied Biosystems). The *COI* and *white* gene sequences were aligned first by nucleotides using the Muscle algorithm [27] implemented in SeaView [28] and then by amino acid using TranslatorX [29].

The ITS2 sequences were annotated for the 5.8S and 28S ends using the ITS2 annotation tool [30] in the ITS2 Database [31]. ITS2 secondary structure was then predicted for each sequence using Mfold [32] and the sequence that gave the lowest minimum free energy, ΔG, was used as a template to model the secondary structure of sequences using the Custom Modeling tool at the ITS2 Database. Sequences with secondary structures were then aligned and edited in 4Sale [33,34]. Sequence edits were performed in Bioedit [35].

### Phylogenetic analysis

Bayesian analysis was applied to *COI*, ITS2, *white* and combined gene sequence data using partitioning schemes to allow different partitions to have their own model characteristics (composition, rate matrix and among-site variation) and to allow for among-partition rate variation. Optimal evolutionary models were determined for each partition using the Akaike Information Criterion (AIC) in jModelTest 2 ([36]; Additional file 1). Optimal partition schemes were calculated using Bayes factors [37]. All Bayesian analyses were performed using MrBayes [38] on Bioportal [39] and each analysis consisted of two simultaneous runs, which were then repeated to provide confirmation of convergence of posterior probability distribution. While all ITS2 clones were included in the isolated gene analysis, only a single randomly selected ITS2 clone from each individual was included in the combined gene analysis.

For all Bayesian analyses, each run was 12 million generations long and the first six million were discarded as burn-in. The Metropolis-coupled Markov chain Monte Carlo strategy was used with six heated chains; adequate mixing was achieved by setting the chain temperature to between 0.1 and 0.2. Convergence of topology between the two runs was monitored using the average standard deviation of split frequencies - this index consistently fell to below 0.015 in the post-burn-in samples. Convergence was also monitored by noting the potential scale reduction factor values - these values were all approximately 1.0 in the post-burn-in samples. Consensus trees were constructed containing nodes with posterior probability support greater than 70%. Trees were drawn using the R package APE [40].

### Barcoding analysis

Individual pairwise Kimura-two-parameter (K2P) [41] distance matrices were constructed for *COI*, ITS2 and combined *COI*-ITS2 using APE. All ITS2 clones were included in this analysis, and these were combined with the corresponding *COI* sequence for each individual in the combined *COI*-ITS2 dataset. K2P Neighbor Joining (NJ) trees were constructed using Mega [42], with 10,000 bootstrap replicates. Minimum inter-specific and maximum intra-specific distances for each individual was calculated using the R package SPIDER [43]. The utility of these genes for barcoding was further tested using the “Best Close Match” (BCM) algorithm in TaxonDNA v1.7.8 [44]. This algorithm involves matching the query sequence to the most similar barcode within a specified species threshold. The query is then assigned the species name if it is within the 95th percentile of all intraspecific distances. The use of such a threshold offers advantages over arbitrary species identification thresholds as it is rigorously derived and can account for differences in mutation rate among loci and divergence among taxa.

## Results

### Phylogenetic analysis

A total of 61 individuals from the Strodei Subgroup were included in the analysis. After alignment these yielded 53 unique *COI* sequences of 638 base pairs in length, 49 unique ITS2 sequences of 432 base pairs in length, and 57 unique *white* sequences of 716 base pairs in length (including the intron of 109 base pairs in length). This gave a combined data set of 61 unique sequences of 1786 base pairs in length. *Anopheles kompi* Edwards (*COI* and *white* GenBank accession no. JF923715 and JN413731, respectively), *Anopheles lutzii* Cruz (*COI* and *white* GenBank accession no. JF923668 and JN392485, respectively), and *Anopheles galvaoi* Causey (*COI*, ITS2 and *white* GenBank accession numbers were KC330264, KC330295 and KC330337, respectively) were used as outgroup taxa. *Anopheles kompi* and *An. lutzii* could not be aligned at the ITS2 locus. The ITS2 locus was left un-partitioned for the Bayesian analysis, whereas, the best partition schemes for *COI* and *white* were those that partitioned by codon position with among-partition rate variation. The best partition scheme for the combined locus dataset was one that partitioned by locus and codon position.

**Figure 1:**
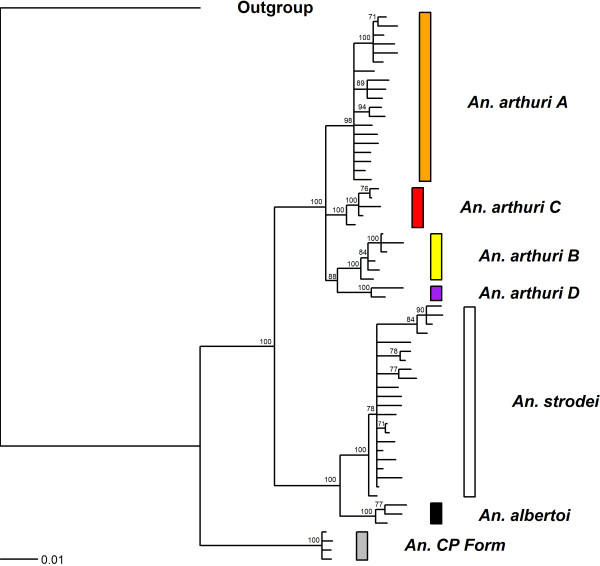
**Bayesian tree of combined *COI*, *white *and ITS2 sequences from the *Anopheles strodei *subgroup.** The data were partitioned by gene and codon. Numbers at branches indicate Bayesian posterior probability (≥ 70%). *Anopheles galvaoi* was included as an outgroup.

**Figure 2:**
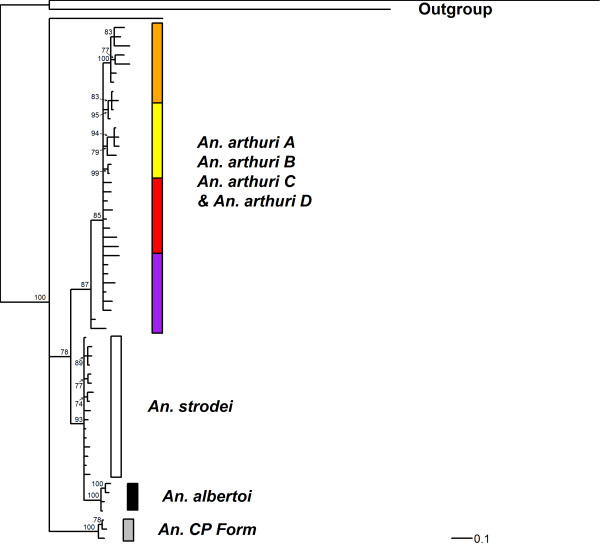
**Bayesian tree of white sequences from the *Anopheles strodei *subgroup.** The data were partitioned by codon position with among partition variation. Numbers at branches indicate Bayesian posterior probability (≥ 70%). *Anopheles kompi, Anopheles lutzii* and *Anopheles galvaoi* were included as outgroup taxa.

**Figure 3:**
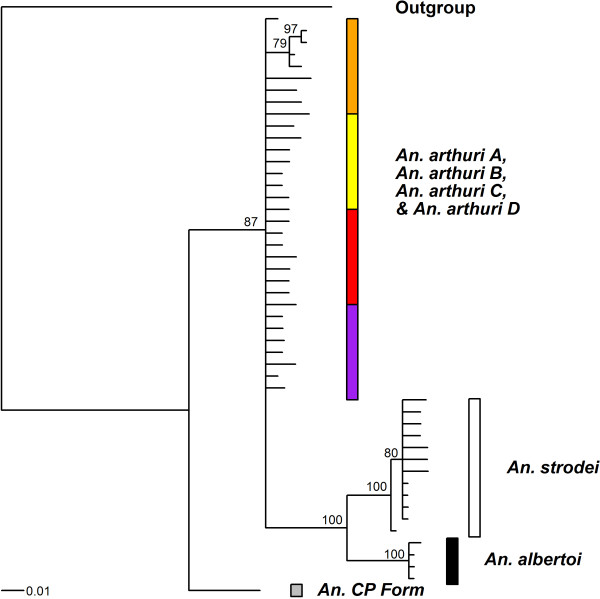
**Bayesian tree of ITS2 sequences from the *Anopheles strodei *subgroup.** Numbers at branches indicate Bayesian posterior probability (≥ 70%). *Anopheles galvaoi* was included as an outgroup.

**Figure 4:**
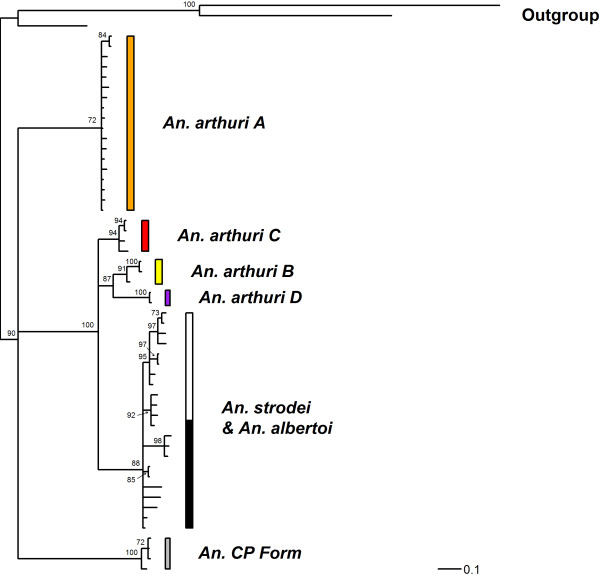
**Bayesian tree of COI sequences from the *Anopheles strodei *subgroup.** The data were partitioned by codon position with among partition variation. Numbers at branches indicate Bayesian posterior probability (≥ 70%). *Anopheles kompi*, *Anopheles lutzii* and *Anopheles galvaoi* were included as outgroup taxa.

**Figure 5:**
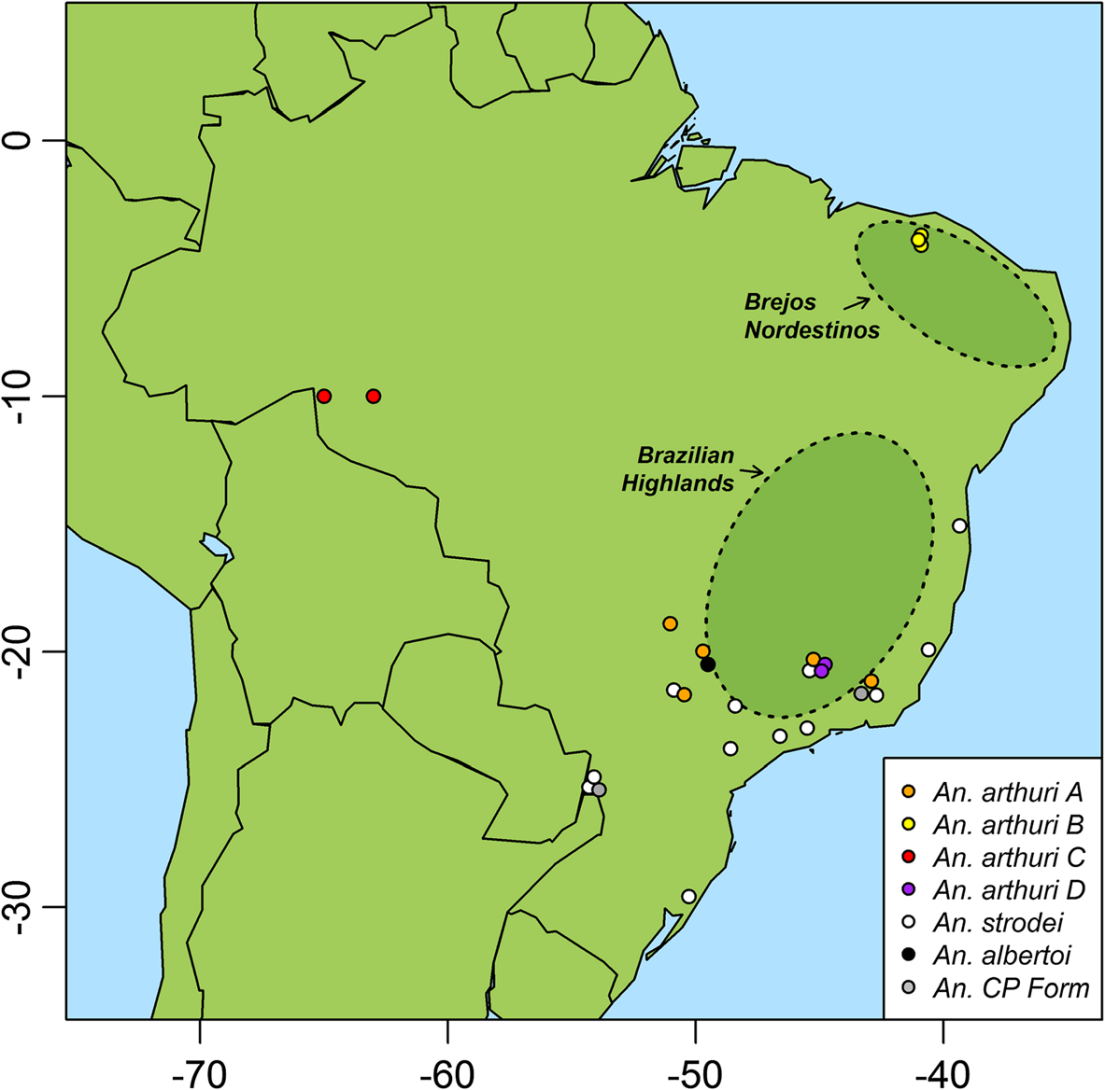
**Sample distribution map.** Geographical coordinates are taken from Table 1 and species are defined according to clades obtained from the Bayesian analysis.

Results of Bayesian analyses showed support for six clades in the combined gene tree (Figure 1). *Anopheles* CP Form was resolved from all other individuals across all gene trees. In the *white* gene (Figure 2), it was found as a sister to one of the outgroup taxa (*An*. *galvaoi*) and to a clade containing the remaining *An. strodei* subgroup. *Anopheles arthuri* s.l. individuals were resolved from others across all gene trees (Figures 1, 2, 3, and 4). There was no evidence for divergence among *An. arthuri* s.l. individuals at ITS2 and *white* genes, and at the ITS2 locus there was intra-genomic variation. Individuals that required cloning yielded between 2 and 6 clones and this intra-genomic variation (0.26% - 1.09% K2P) frequently exceeded inter-genomic variation. However, *An. arthuri* s.l. was resolved into four geographically meaningful clades in the *COI* gene tree (Figure 4). These four clades were found across Brazil (Figure 5), in the central/southern Brazilian states of Goiás, Minas Gerais and São Paulo (72% Bayesian Posterior Probability, BPP; herein denoted *An. arthuri* A), the northern state of Ceará (91% BPP; denoted *An. arthuri* B), the western Amazonian state of Rondônia (94% BPP; denoted *An. arthuri* C) and southern Minas Gerais state (100% BPP; denoted *An. arthuri* D), with the last being a sister to the Ceará clade (87% BPP). *Anopheles* CP Form, *An. albertoi* and *An. arthuri* s.l. can be resolved from *An. strodei* individuals at ITS2, *white* and combined gene trees. However, *An. strodei* and *An. albertoi* form a single clade at the *COI* gene tree (88% BPP).

### Barcoding analysis

**Figure 6:**
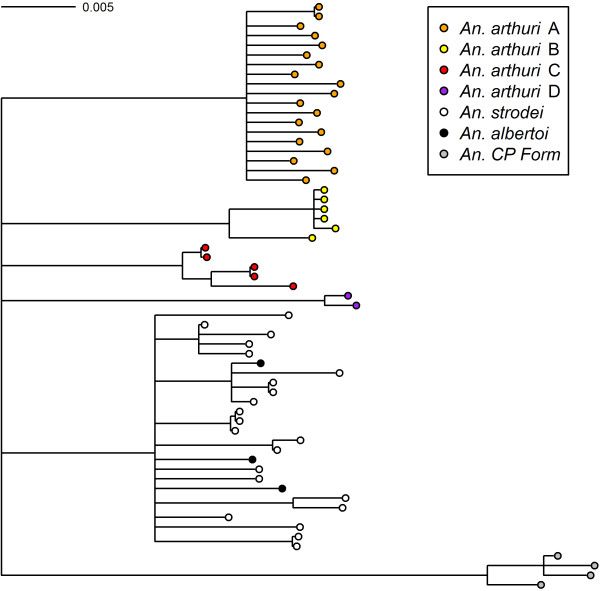
**Bootstrapped neighbor joining tree of COI sequences from the Anopheles strodei subgroup.** Constructed with Kimura’s two parameter (K2P) distances and supported by 10,000 bootstrap replicates. All clades have greater than 70% bootstrap support.

**Figure 7:**
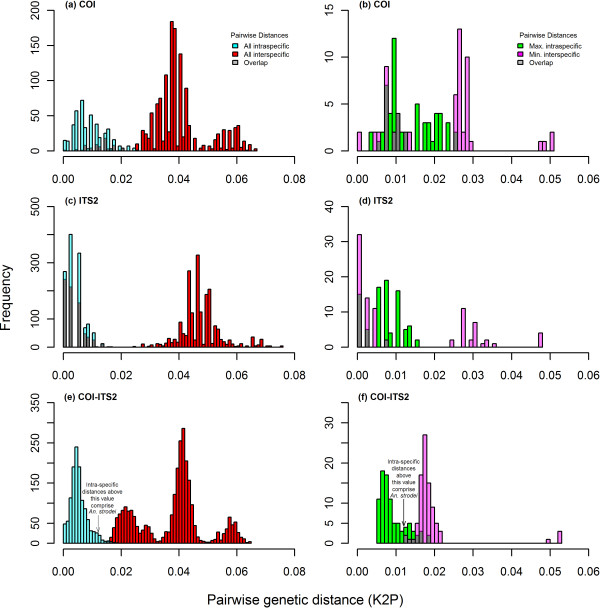
**Frequency distribution of intraspecific and interspecific genetic divergence in the *Anopheles strodei *subgroup.** The distributions in (**a**), (**c**) and (**e**) contain all intraspecific and interspecific pairwise genetic distance of specimens described in Table 1. The distributions in (**b**), (**d**) and (**f**) contain only maximum intraspecific and minimum interspecific pairwise genetic distance for each individual. Pairwise genetic distances were calculated using Kimura’s two parameter (K2P) distance.

The Barcode NJ tree for *COI* (Figure 6) shows six clear groups. Individuals from *An. arthuri* s.l. can be found in the same four separate groups as found in the phylogenetic analysis. Figure 7 (a) shows a histogram of all intra- and inter-specific K2P *COI* differences among individuals and Figure 7 (b) shows a histogram of maximum intra- and minimum inter-specific K2P *COI* differences among individuals, when ordered into clades as defined by the phylogenetic analysis. Distances are measured in 0.001 (0.1%) intervals. There are no barcoding gaps present in either histogram, and the intra- versus inter-specific distances shows a very high degree of overlap.

**Figure 8:**
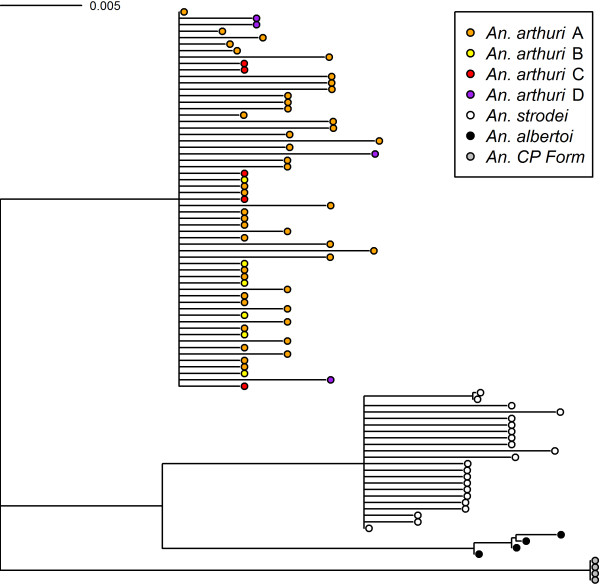
**Bootstrapped neighbor joining tree of ITS2 sequences from the *Anopheles strodei *subgroup.** Constructed with Kimura’s two parameter (K2P) distances and supported by 10,000 bootstrap replicates. All clades have greater than 70% bootstrap support.

The Barcode NJ tree for ITS2 (Figure 8) shows four clear groupings – *An*. *arthuri* s.l., *An*. CP Form, *An*. *albertoi*, and *An*. *strodei*. Figure 7 (c) and (d) show histograms of all intra- and inter- specific K2P ITS2 distance among individuals, and maximum intra- and minimum inter-specific K2P ITS2 distances among individuals, respectively, when ordered into clades as defined by the phylogenetic analysis. Again, there are no barcoding gaps present, and the intra- versus inter-specific distributions shows a very high degree of overlap.

The BCM analyses further explored the intra- and inter-specific distances in the *COI* (Additional file 2) and ITS2 (Additional file 3) barcodes. Threshold values for 95% of all intra-specific distances were determined for each barcode to evaluate whether a query (matching a test sequence to a reference sequence) had a close enough barcode match for identification. These were 1.92% for *COI* and 1.06% for ITS2. In total, 91.80% (n = 56) of queries were correctly identified by the *COI* barcode according to the BCM criteria. The *COI* barcode was highly effective at correctly identifying queries from *An.* CP Form, *An. arthuri* A, *An. arthuri* B, *An. arthuri* C, and *An. arthuri* D. All queries from these five species were successfully matched to their respective species groups. However, all three queries from *An. albertoi* and two from *An. strodei* were not successfully matched. The three *An. albertoi* queries were incorrectly matched to *An. strodei*, the first *An*. *strodei* query was incorrectly matched to *An*. *albertoi* and the second *An*. *strodei* query was ambiguous as it was matched equally to both *An*. *albertoi* and *An*. *strodei*. The highest levels of intraspecific distances among all seven species were consistently from *An. albertoi* and *An. strodei*. Although intraspecific comparisons in the study ranged from 0% to 2.58%, all of the intraspecific comparisons above 1.27% (n = 232) were among *An. albertoi* and *An. strodei COI* barcodes and intraspecific comparisons above 2.00% (n = 32) were solely from *An. strodei COI* barcodes.

The BCM analysis for the ITS2 barcode found that only 59.55% (n = 53) of queries were correctly identified. All *An.* CP Form, *An. albertoi and An. strodei* queries were correctly matched to their respective species. However, 39.32% (n = 35) of queries were ambiguous and 1.12% (n = 1) were incorrect and these came entirely from the *An. arthuri* species.

**Figure 9:**
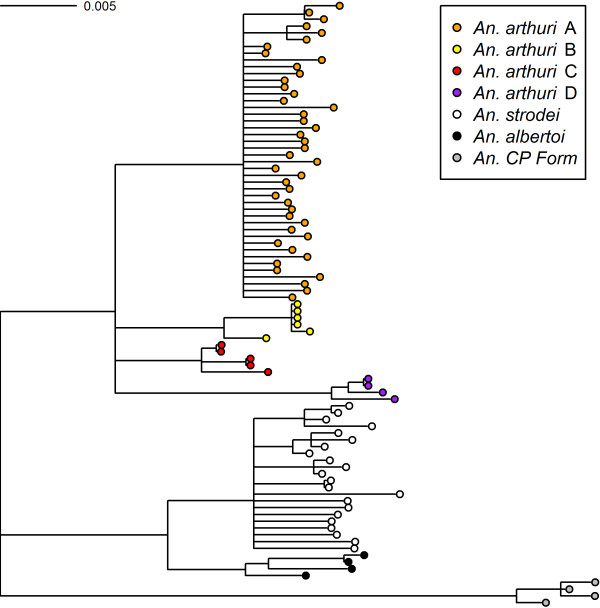
**Bootstrapped neighbor joining tree of COI and ITS2 sequences from the *Anopheles strodei *subgroup.** Constructed with Kimura’s two parameter (K2P) distances and supported by 10,000 bootstrap replicates. All clades have greater than 70% bootstrap support.

The *COI* barcode, therefore, correctly identified all *An.* CP Form, *An. arthuri* A, *An. arthuri* B, *An. arthuri* C, and *An. arthuri* D, while the ITS2 barcode correctly identified all *An.* CP Form, *An. albertoi* and *An. strodei* individuals. A combined *COI*-ITS2 barcode was therefore tested first using a NJ tree (Figure 9) and then using the BCM analysis (with a 95% intraspecific variation threshold of 1.11%; Additional file 4). The results showed that all species could be resolved using the NJ tree and all BCM queries successfully identified *An.* CP Form, *An. arthuri, An. strodei, An. arthuri* A, *An. arthuri* B, *An. arthuri* C, and *An. arthuri* D. This was despite maintaining a small degree of overlap between intra- and inter-specific distances due to inflated levels of genetic variation in *An*. *strodei* (Figure 7 (e) and (f)).

## Discussion

A recent study has added two additional species (*An. albertoi* and *An. arthuri*) to the *An. strodei* subgroup [12]. It also found support for a distinct morphological form, referred to as “CP Form”, based on a single individual captured in the state of Paraná. In the current study we identified seven distinct lineages, of which three represented currently recognized species (*An*. *strodei*, *An*. *arthuri* s.s./*An. arthuri* A and *An*. *albertoi*), and four are undescribed (*An*. *arthuri* B, *An*. *arthuri* C, *An*. *arthuri* D and *An*. CP form).

The first important observation of the phylogeny is several incongruences among topologies generated from the DNA sequences. While ITS2 resolves *An. strodei* and *An. albertoi*, it fails to identify lineages within *An. arthuri* s.l. The *COI* region, however, clearly resolves four *An. arthuri* s.l. lineages, but fails to resolve *An. albertoi* and *An. strodei*. Differences between the gene genealogies and the species genealogy could be the result of incomplete lineage sorting or, in the case of ITS2, incomplete concerted evolution. In relation to incomplete lineage sorting, ancestral haplotypes can be retained in cases of recent speciation and/or large breeding populations, potentially resulting in the obscuring of phylogenetic signal among species. This process may explain the inability to resolve *An. strodei* and *An. albertoi* at the *COI* gene. Incomplete concerted evolution occurs when the rate of homogenization among copies in the ITS2 multi-gene family is insufficient to bring about fixation, potentially resulting in intra-genomic variation and shared haplotypes among closely related species. This process appears to be the cause of high levels of intra-genomic variation in several species of *Anopheles*[45-49] and can potentially blur phylogenetic signal in some species, as appears to be the case among the *An*. *arthuri* s.l. lineages in the current study.

Our phylogenetic analysis supports distinction of *An. albertoi* and *An. arthuri* s.l. as in previous work [12], but also further splits *An. arthuri* s.l. into four distinct lineages (at the *COI* and combined gene tree). These lineages are geographically and ecologically distinct, and are herein referred to as *An. arthuri* A (from a central/southern Brazilian region of Goiás, Minas Gerais, and São Paulo), *An. arthuri* B (from the northern Brazilian state of Ceará), *An. arthuri* C (from the Amazonian state of Rondônia) and *An. arthuri* D (from southern Minas Gerais). The *An. arthuri* A lineage can be found in the Interior Forest Subregion of the Atlantic Forest, where seasonal semi-deciduous forest dominates [50]. Individuals from this lineage were found on both the western and eastern slopes of the Brazilian Highlands (Figure 5). Three of these individuals (MG07_1_100, MG07_10_106 and MG07_18_100) were previously included in an assessment of egg morphology using scanning electron microscopy [12] and were found to be representative of the *An. arthuri* type specimen. It is therefore likely that *An. arthuri* A identified in this study is representative of *An. arthuri* s.s. The *An. arthuri* B lineage is found in the Brejos Nordestinos Subregion of the Atlantic Forest. This subregion marks the extreme northern reach of the Atlantic Forest and consists mainly of seasonal semi-deciduous forest or dense ombrophilous forest “islands” covering isolated plateaus, which are surrounded by arid Caatinga lowlands [50]. Whereas the Atlantic Forest was until recently largely contiguous, the forests of Brejos Nordestinos were isolated much earlier, during the climatic cycles of the Pleistocene [51]. Populations from these forest islands are therefore likely to be subject to greater levels of divergence via genetic drift and barriers to gene flow. The *An. arthuri* C lineage is found in the southern reaches of the Amazonian river basin, to the north and west of the Parecis Mountains. We found no evidence for the presence of *An*. *strodei* in this region and that it is likely that previous reports of *An*. *strodei* found naturally infected with *Plasmodium vivax* in Rondônia [23] actually may refer to *An*. *arthuri* C. The ranges of *An. arthuri* A, *An. arthuri* B and *An. arthuri* C lineages are thus ecologically divergent, and appear to be highly allopatric (lineage sampling localities separated by more than 1600 km). Two individuals also exist which were collected from Oliveira in the state of Minas Gerais with *COI* haplotypes that are significantly distinct from all others in the complex (>2.92% variation). These individuals were collected from a site in the Rio Pará Valley, near the headwaters of the São Francisco and the Paraná Rivers, at an altitude of approximately 1,000 meters, in a largely un-forested landscape at the interface of Brazil’s Atlantic Forest and Cerrado eco-regions. They are found locally sympatric with *An. strodei* and *An. arthuri* A in this mountain valley but their absence from all other localities indicates that this species may be confined to mountainous areas in the Brazilian Highlands. Their distinction from other species may have been shaped by the considerable topographical structure in this region, serving as a barrier to gene flow and isolating them from other populations, and the varying selective pressures that potentially exist across the enclosed humid habitat of the Atlantic Forest and the open dry habitat of the Cerrado. These distinct Rio Pará Valley haplotypes are, therefore, tentatively identified as *An. arthuri* D, but clearly further sampling in more northerly localities in the São Francisco Valley is required to determine whether this represents a distinct species.

Previous analysis of the *An. strodei* subgroup found that *An. albertoi* can be distinguished morphologically, from its sister species by differences in the eggs (absence of a float) and male genitalia, and genetically, with the *white* and combined *white*-*COI* genes [12]. Using *An. albertoi* individuals from the study of Sallum *et al.*[12], we again differentiated this species from *An*. *strodei* and provide further genetic support for this lineage at the ITS2 gene. We have found the distribution of this species straddles the Brazilian Highlands, with individuals identified from the coastal forest of Serra do Mar in the state of São Paulo and the interior forest of the state of Minas Gerais, where it is found locally sympatric with *An. arthuri* A. The sampling associated with *An. strodei* is the most extensive among species in the study. Samples came from 14 different localities in six Brazilian states, some of which are separated by more than 2,000 km. Although there was genetic and morphological support for this species, the substantial range of intra-specific distance at *COI* (0–2.58%) can be contrasted with intra-specific distances found in other species in this study (all less than 1.59%) and the 1% species identification threshold proposed in Ratnasingham and Hebert [3]. Comparable data, i.e. intra-specific pairwise distance ranges, from other studies of *Anopheles* species are scant, but higher intra-specific *COI* distances have been observed across a range of well supported species from the butterfly family Lycaenidae Leach [8]. Although the distribution of *An. strodei* haplotypes does not demonstrate geographic partitioning and there is no apparent variation in morphology or habitat, the levels of intra-specific variability present may be indicative of a high degree of cryptic population genetic structure. A comprehensive population genetic study, which includes more samples (n > 20) from each of the 14 *An. strodei* localities detailed here, would help address this question and lead to a better understanding of the nature of genetic variation in this species.

The *An*. CP Form individuals have previously been resolved from other species in the *An*. *strodei* subgroup based on differences observed in the male genitalia of a single individual collected in Foz do Iguaçu in the state of Paraná [12]. In the current study we have included additional individuals morphologically identified as *An*. CP Form from Coronel Pacheco in the state of Minas Gerais and have found that all CP Form individuals can be resolved genetically across multiple genes. Although the *An*. CP Form collection sites (Foz do Iguaçu, Paraná and Coronel Pacheco, Minas Gerais) are confined to the Interior Forest subregion of the Atlantic Forest, they are highly disparate, separated by more than 1,500 km. This lineage’s geographic distribution is further extended by its identification in the coastal state of Espírito Santo [52]. In addition, the lineage is found locally sympatric with other species from the *An. strodei* subgroup, namely *An. strodei* in the west, and both *An. strodei* and *An. arthuri* A in the east.

Generally, the most closely related species in the complex, i.e. within the *An. strodei*/*An. albertoi* clade and within the *An. arthuri* clade, are not found sympatrically, which may indicate allopatric speciation is the most important mode of speciation in this complex. However, the one exception to this pattern is species that are found in Rio Pará Valley. Here we find both *An. arthuri* A and *An. arthuri* D (as well as *An*. *strodei*). It may be that the *An. arthuri* D clade represents a Brazilian Highland endemic as it has been unreported among more southerly and easterly localities, and that the southern limits of its range overlap with the northern limits of its sister species. However, further sampling through more northern localities of the São Francisco Valley and Brazilian Highlands is necessary to identify the breeding range of these species.

No single barcode was found to be effective at resolving all species identified from the phylogenetic analysis of the *An. strodei* subgroup. Neither *COI* nor ITS2 alone proved to be reliable as barcodes, largely because of their inability to resolve *An*. *albertoi*/*An*. *strodei* and *An*. *arthuri* species, respectively (as is evidenced by the considerable overlap between intra- and inter-specific differences). Many barcoding studies have demonstrated that the existence of substantial barcoding gaps permits effective species identification and discovery [7,53,54]. In closely related species, such as those found in species complexes, overlapping intra- and inter-specific variation are more likely and mainly due to processes such as incomplete lineage sorting [55]. However, although identification success generally declines with increasing overlap between intra- and inter-specific distances, studies have also shown that the existence of the barcoding gap does not predict the identification success of DNA barcoding [56,57]. In the current study we found that, although the *COI* and ITS2 barcodes do not have a barcoding gap and exhibit considerable overlap among the species identified through phylogenetic and morphological analysis, a combined *COI*-ITS2 barcode reduced the extent of overlap and provided a useful tool for species identification in the complex. An important advantage that the *COI* barcode has over the ITS2 barcode is the relative ease with which it can be aligned. The ITS2 barcode is highly variable in relation to indels, and alignment of ITS2 sequences in *Anopheles* becomes extremely difficult in any other species other than close relatives. Therefore, while the *COI*-ITS2 barcode may provide an effective species tool in other anopheline species complexes, ITS2 sequence alignment is a mitigating factor for its use in more distantly related species.

Several studies have demonstrated that the extent and scale of intra-specific sampling and the inclusion of closely related species can have a significant impact on the global application of barcodes [58-60]. While intra-specific variation will tend to increase with increased geographical sampling, due to isolation by distance and geographic structure, inter-specific variation will tend to decrease due to the inclusion of more closely related allopatrically distributed species [61]. The current study has attempted to sample from a diverse range of localities from across the complexes’ distribution (in nine Brazilian states) but most of the newly and tentatively identified species are clearly under-represented, numerically and geographically, particularly in the case of *An. albertoi* (n = 3) and *An. arthuri* D (n = 2). Also, although *An. arthuri* C is better represented in the study than the previous two species, the geographic distribution of these samples is quite limited versus potential *An. arthuri* C breeding habitat in the Amazon basin. Recent studies have found that sample sizes used in DNA barcoding are generally low [60,61] and that a sampling strategy of less than 20 individuals per species is unlikely to adequately represent intra-specific variation [60]. The shortcomings of the current study can therefore be addressed by future sampling in the geographically disparate localities, particularly within the Brazilian Highlands and the Amazon basin.

## Conclusion

We identified seven possible species in the *Anopheles strodei* subgroup, three of which are reported here for the first time. The role of these as potential vectors of malaria is largely unknown but *An*. *strodei* individuals previously found naturally infected with *Plasmodium vivax* in the Amazon region are likely to be *An*. *arthuri* C identified herein. We found poor support for the use of a single barcode for species identification in this Subgroup. Although single barcodes may be useful to estimate minimum levels of speciosity in complexes, we found significant numbers of ambiguous or incorrect query matches when using this approach and would caution against their use for effective species identification in Anopheline species complexes. Instead, we propose a combined *COI*-ITS2 barcode as a potentially useful tool for species identification in the *An. strodei* complex, but recommend further sampling of intra-specific variation in order to more effectively assess the utility of this multi-locus barcode.

## Competing interests

The authors declare that they have no competing interests.

## Authors’ contributions

MAMS and BPB conceived and designed the experiments. TMPO carried out the molecular laboratory work. ESB and MAMS did the field collections and identified the specimens. BPB performed the data analysis. BPB wrote the paper, with contributions from MAMS. All authors read and approved the final manuscript.

## Supplementary Material

Additional file 1 Models used for locus and codon positions. Click here for file

Additional file 2 Identification of species based on “best close match” and COI sequences. Click here for file

Additional file 3 Identification of species based on “best close match” and ITS2 sequences. Click here for file

Additional file 4 Identification of species based on “best close match” and combined COI-ITS2 sequences. Click here for file
